# New Strategies for the Treatment of Phenylketonuria (PKU)

**DOI:** 10.3390/metabo4041007

**Published:** 2014-11-04

**Authors:** Pietro Strisciuglio, Daniela Concolino

**Affiliations:** 1 Department of Pediatrics, University “Federico II” of Naples, Naples 8823100, Italy; 2 Department of Pediatrics, University “Magna Graecia” of Catanzaro, Catanzaro 88100, Italy; E-Mail: dconcolino@unicz.it

**Keywords:** Glycomacropeptide (GMP), large neutral amino acids (LNAA), Phenylketonuria (PKU), tetrahydropterin, phenylalanine ammonia-lyase (PAL)

## Abstract

Phenylketonuria (PKU) was the first inherited metabolic disease in which dietary treatment was found to prevent the disease’s clinical features. Treatment of phenylketonuria remains difficult due to progressive decrease in adherence to diet and the presence of neurocognitive defects despite therapy. This review aims to summarize the current literature on new treatment strategies. Additions to treatment include new, more palatable foods based on glycomacropeptide that contains very limited amount of aromatic amino acids, the administration of large neutral amino acids to prevent phenylalanine entry into the brain or tetrahydropterina cofactor capable of increasing residual activity of phenylalanine hydroxylase. Moreover, human trials have recently been performed with subcutaneous administration of phenylalanine ammonia-lyase, and further efforts are underway to develop an oral therapy containing phenylanine ammonia-lyase. Gene therapy also seems to be a promising approach in the near future.

## 1. Introduction

**Figure 1:**
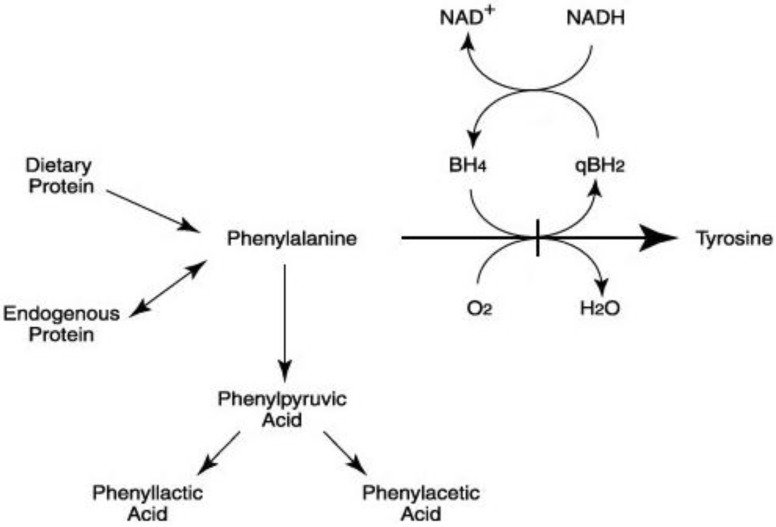
Metabolic pathway of phenylalanine.

Phenylketonuria (PKU OMIM 261600) is an autosomal recessive disorder caused by mutations in the phenylalanine hydroxylase (PAH) gene. It results in the accumulation of phenylalanine (Phe), an essential amino acid mainly metabolized in the liver by the phe hydroxylase (PAH) system. This enzyme hydroxylates Phe to tyrosine (Figure 1) requiring tetrahydrobiopterin (BH4) as a co-factor. Defects in either PAH or the production or recycling of BH4 may result in hyperphenilalaninemia which can cause intellectual disability if untreated [1].

The natural history of the phenylketonuria consists in a progressive irreversible neurological impairment during infancy and childhood. The most common outcome is severe mental retardation often associated with a “mousy” odor, eczema and reduced hair skin and iris pigmentation; also reduced growth, microcephaly and neurological signs as tremor, epilepsy are present. All untreated patients have behavioral problems as hyperactivity, stereotypy and anxiety. The severity of the clinical phenotype directly correlates with blood phenylalanine levels that reflect the degree of enzymatic deficiency.

Neonatal screening by measuring Phe levels in blood spots on filter paper can be identify affected infants at birth. Early treated PKU patients have normal intellectual quotients (I.Q.), but can have an I.Q. gap when compared to their non-PKU siblings [2]. In addition, patients with PKU frequently have lower scores for certain neuropsychological functions, with executive function being the most affected area [3].

The prevalence of PKU varies by country ranging from between one in 10,000 and one in 20,000 births in U.S.A. and Europe [4]. Depending on the genotype and severity of the enzyme defect, various forms of PKU with different clinical outcomes have been described [5]. These can be classified on the basis of blood Phe levels at diagnosis and dietary Phe tolerance. Until recently, a strict low-Phe diet was the only therapy available. The PKU diet consists of are striction of natural proteins in the diet and supplementation with special medical formulas that supply vitamins, minerals, and all essential amino acids except Phe. Dietary treatment has been very effective in the prevention of impaired cognitive development, but still has its shortcomings. Growth delay and specific deficiencies of calcium, zinc, selenium, iron, and vitamin B12 were reported with the early formulas [6,7,8]. The PKU diet is also cumbersome for the patients and their families, often leads to a lack of compliance starting in childhood. For these reasons, new medical formulas with improved nutritional quality and palatability have been developed to lower the volume of food and to improve compliance.

This review discusses the evolution of dietary treatment leading to improved nutritional management using an increased variety of PKU formulas, some of which containing glycomacropeptide, the introduction of new formulations of Large Neutral Amino Acid (LNAA) supplementation and pharmacological therapy with tretahydrobiopterin cofactors and enzyme substitution therapy.

The review was based on literature search on PUBMED for articles between 2000 and 2014 with the term “phenylketonuria” and “PKU” in combination with the following terms “diet”, “enzyme therapy”, “gene therapy”, “glycomacropeptide”, “large neutral amino acids”, “sapropterin”, “tetrahydrobiopterin” and “chaperones”. Only publications in English were taken in consideration.

## 2. Dietary Treatment

Treatment of phenylketonuria is still not optimal, and so novel alternative formulas have been sought. New dietary therapies include more palatable formulas with improved caloric content allowing better compliance [9]. Generally the diet has inadequate amounts of taurine and other micronutrients derived from animal products. In addition, the diet is low in long-chain poly unsatured fatty acids (LC-PUFAs) such arachidonic acid (AA) and docosahexaenoic-acid (DHH). Many formulas are now supplemented with long-chain poly unsatured fatty acids after studies have shown that LC-PUFAs improve the maturation of the visual system and motor skills in patients with PKU [10,11]. Synthetic amino acids are the primary protein source in the nutritional management of PKU. However, di-tripeptides are more effectively absorbed from the intestine. Glycomacropeptide (GMP) is a naturally occurring protein derived from cheese whey, which is low in phenylalanine and an excellent source of protein for PKU patients. GMP improves the taste, variety, satiety [12], and convenience of the diet with consequent improved dietary compliance, metaboli ccontrol, and finally a better quality of life for patients [13]. GMP is an intact protein source and, compared to free amino acids in standard formulas, improves protein retention and phenylalanine utilization [13]. One reason for non-compliance is the financial burden that many families face with formula and low protein foods because in many countries, as in the USA, not all insurance companies are required to pay for formula or cover low protein foods. Recent efforts to improve adherence to dietary treatment by restricting dietary protein have shown to be an alternative way of maintaining metabolic control in PKU [14]. Overall, restriction of the intake of natural protein with supplementation of a Phe-free medical formula remains the cornerstone of PKU management and is being helped by the developments of a larger variety of medical foods, some of which made with GMP, a low-phe whey protein.

Dietary treatment is monitored by frequent measurement of plasma/blood phenylalanine levels. A portable Phe monitoring device could improve dietary adherence by providing Phe levels in real time and, thereby, provide immediate motivation to patients with PKU [15]. Unfortunately, nobody has developed such a device up to this point in time. Iontophoretic extraction of Phe from the skin correlated with plasma levels of Phe above 300 µmol/L. but was not sufficiently sensitive to detect low phenylalanine levels [16].

## 3. Large Neutral Amino Acid Supplementation

**Figure 2:**
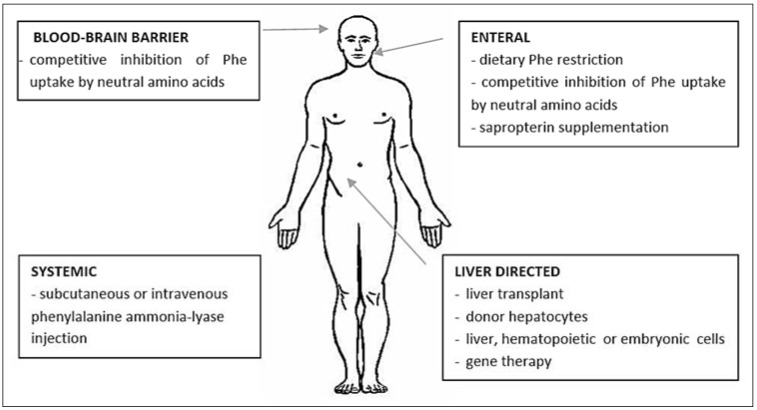
Potential therapeutic approaches for phenylketonuria (PKU) (modified from Harding, C.O., *et al*.)

Other novel therapeutic approaches can be categorized by the site of action or target organ (Figure 2) [17]. These categories include enteral, systemic, liver-directed approaches. Dietary restriction of Phe intake is an example of enteral approach. Alternatively Large Neutral Amino Acid (LNAA) can be used. LNAA can compete with the same transporter of Phe across the gastrointestinal and blood brain barrier to reduce Phe absorption and entry into the brain [18]. A double blind, placebo-controlled study indicated a significant decline in blood Phe concentration in patients with PKU treated with LNAA for 2 weeks suggesting that LNAA compete with the transport of Phe in the gastrointestinal trac [19]. These studies suggest that adding LNAA to the diet of patients with PKU could reduce blood Phe concentrations.

Oral LNAA supplementation can reduce brain Phe concentrations and improve neuropsychological functioning [20]. Differences in outcome may be related to composition, dosing route of administration, and duration of the supplementation period. Reduced brain LNAA concentrations have been reported in PKU mice [21], and restoring levels of large neutral amino acids in the brains may improve cognitive outcome in PKU. Hoeksma *et al* [22] demonstrated a significant negative correlation between plasma Phe concentration and cerebral protein synthesis in patients with PKU. This leads to development of new medical foods with higher concentrations of LNAA and fortification with vitamins and lutein, an antioxidant important for the development of the brain [23]. Studies in PAH ^enu2^ mice provide support for the use of a variety of non-physiological amino acids to act as competitive inhibitors of brain transporters to reduce brain Phe concentrations with minimal impact on other down-stream intermediates [24].

The evidence to support the efficacy of LNAA supplementation to significantly reduce blood Phe levels in patients with PKU is still limited. The effects of LNAAs have been assessed only for short times and in a limited number of patients using variable dosages (250–1000 mg LNAA/Kg/day) and different formulations of LNAA [25].Patients with Phe levels above 1000 µmol/L had a~40% of decrease in plasma Phe levels [25]. One randomized controlled study reported a positive effect on executive functions [26].

In summary, LNAA supplementation either alone or in combination with a low-Phe diet has been shown to improve health outcome for individuals unable to follow the low Phe diet. However long term outcome studies assessing efficacy and safety of LNAA supplementation are needed.

## 4. Tetrahydropterin as Enzyme Enhancement Therapy for PKU

Some patients with PKU respond to pharmacological doses of tetrahydropterin (BH4) with reduced blood Phe levels as first shown in 1999 by Kure *et al.* [27]. At pharmacological doses, sapropterin hydrochloride acts as a molecular chaperone that promotes correct folding and stability of the PAH enzyme [28]. The recommendations on how to test patients with hyperphenylalaninemia for BH4 responsiveness are evolving [29,30,31]. All patients with Phe levels>360 µmol/L should be tested for responsiveness to sapropterin (20 mg/Kg/day). Multiple Phe levels need to be obtained at baseline and after starting BH4 to account for normal physiological variation sin Phe levels. The effect of BHA is evaluated after ashort-term (up to 48 h) [32] and long-term (up to several weeks) [33] to demonstrate consistent reduction of Phe levels as compared to baseline. A decrease in blood Phe of 30% or more from baseline indicates response to sapropterin therapy [34]. Patients with milder phenotype generally are responsive [35]. Long-term treatment with sapropterin of responsive patients with PKU improves Phe tolerance and, in some cases, allows them to discontinue restrictive diets [36,37,38].

In summary, treatment with sapropterin resulted in significant (at least 30%) and sustained reductions in blood Phe concentrations and increased dietary Phe tolerance in responsive PKU patients. This has been tested in adults and children with phenylketonuria (Long-term developmental progression in infants and young children taking sapropterin for phenylketonuria: a two-year analysis of safety and efficacy [39]. Successful treatment with sapropterin may lead to a relaxation of a Phe restricted diet, although continued monitoring of blood Phe is advisable [40].

The use of pharmacological chaperones to stabilize or promote correct folding of mutant proteins represents a promising new approach in the treatment of many genetic diseases causing protein misfolding. Proteins and small molecules in addition to tetrahydrobiopterin may act as chaperones to assist in the folding of PAH. Pey *et al.* [41] performed a high-throughput ligand screening of over 1000 pharmacological agents and identified four compounds that enhanced the stability of PAH activity. In particular, the administration of low doses of two of these compounds increased PAH activity in mouse liver. Further studies are necessary before these compounds can be used in clinical practice.

## 5. Enzyme Therapy

Enzyme therapy for PKU is another option whereby the harmful increased levels of Phe can be reduced by the introduction of Phe-metabolizing enzymes changing the metabolic phenotype of PKU, regardless of genotype.

Enzyme therapy can be done either by enzyme replacement with PAH or by enzyme substitution with phenylalanine ammonia-lyase [42]. The metabolism of Phe takes place for the most part in the liver, and orthotropic liver transplantation corrects the metabolic phenotype [43]. Liver transplantation is not a treatment option except for unusual PKU patients who need a liver transplant for another disease such as cirrhosis, because of the burden of daily therapy in transplanted patients. In mice, enzyme replacement with PAH-fusion proteins is a promising approach [44].

Enzyme substitution therapy with phenylalanine ammonia-lyase (PAL) appears more promising. It can act as a surrogate for the deficient PAH and converts the excess systemic Phe to trans-cinnamic acid and ammonia. Both pharmacological and physiological principles of therapy have been demonstrated following the use of PAL given orally or by injection in PKU mouse models [45]. The oral route is complicated by proteolytic degradation of the enzyme, which, however, remains active within the gut. Injected PAL is immunogenic and can cause reactions. The conjugation with polyethylene glycol of PAL (PEG-PAL) has been successful to decrease the immune response [46].

Clinical trials to assess the safety and efficacy of multiple repetitive PEG-PAL injections have been performed. Subcutaneous administration of PAL-PEG was safe, well tolerated, and seemed effective at reducing blood Phe in all participants who received the highest dose with a nadir about six days after injection and an inverse correlation between drug and Phe concentrations in plasma [47]. Modifications of oral PEG-PAL to prevent degradation by digestive enzymes have been initiated in the effort to develop an effective oral therapy [48].

## 6. Cell Directed Therapy

Another treatment under investigation involves liver repopulation with PAH-expressing cells after hepatocyte or hematopoietic stem-cell transplantation. Hepatocyte transplantation is under investigation because donor cells, in order to be efficacious, need to have a selective growth advantage over native hepatocytes [49].

Hepatocyte transplantation has been performed in preclinical studies using various animal models as well in humans with metabolic disorders such us urea cycle defects or glycogen storage disorders. This cellular approach could be possible for the permanent treatment of PKU if a selective growth advantage could be achieved for donor hepatocytes [50]. This treatment has been reported to be successful in an animal model with a selective advantage for the donor cells [17]. However, cell-based therapies using stem cells or more differentiated progenitor cells may represent the future of cell transplantation for treatment of metabolic liver diseases such as PKU.

## 7. Gene Therapy

Gene therapy for the treatment of PKU has been the focus of multiple research group over the last two decades. In a mouse model of PKU, important progress has been made by the use of an adenovirus related gene directed into the liver [51].

However liver-directed gene therapy does not lead to a permanent correction of PAH activity. The vector’s genome is not integrated into the hepatocyte’s DNA, and hepatocyte regeneration leads to elimination of episomalr AAV vector genomes. Reinjection of the same serotype vector also leads to its destruction by antibody-mediated immune reactions. Studies of PKU mouse models have also shown that gene therapy can be successfully delivered to non-hepatic tissues such as the muscle [52]. In fact, the insertion into the muscle cells of vectors containing the necessary genes for PAH and tetrahydrobiopterin synthesis resulted in a system that could convert phenylalanine into tyrosine mimicking the role of hepatic phenylalanine metabolism. The improvement in viral vector design has led to human gene therapy trials for other inborn errors of metabolism including α1 antitrypsin deficiency and Canavan disease [53].

Ongoing research by optimization of the direct muscle approach and improved sustained ability in liver-directed gene therapy might lead to human trials in PKU in the next years.

Another approach is based on *in vitro* read-through of PAH nonsense mutations using aminoglycosides [54]. Approximately 10% of patients with PKU carry a nonsense mutation, which results in the premature insertion of a stop codon. In most cases, these mutations result in unstable mRNA that is rapidly cleared by nonsense-mediated RNA decay. In rare cases, an unstable truncated protein is generated. Aminoglycoside antibiotics such as gentamicin and G-418 can promote read-through of stop codons resulting in production of some protein. The efficacy of aminoglycosides was evaluated in an *in vitro* expression system in two mammalian cell lines (COS-7 and HEX 293). The read-through PAH products exhibited enzymatic activity to levels similar to that found in moderate PKU. Unfortunately, none of the mouse models for PKU carries a nonsense mutation to determine whether the level of read-through in the liver is sufficient to restore Phe tolerance. Further studies are necessary to determine the impact of this approach on the therapy for PKU.

## 8. Conclusions

Patients with PKU should still be treated with dietary therapy, but in the long term the introduction of a wide array of new treatment approaches such as more palatable foods based on the use of GMP products or the administration of LNAA or BH4 could decrease the need for phenylalanine restriction in the diet. Patients’ surveys show that GMP foods have improved taste and are preferred to standard formula. Sapropterin is a safe and effective alternative to conventional dietary treatment of responsive PKU patients by stabilizing their blood Phe concentrations and by lowering the burden of the strict low Phe diet. LNAA supplementation has been efficacious in the lessening the need of a strict Phe diet reduction. Human trials are also underway using an enzymatic approach (PEG-PAL) while preclinical work seems promising in the fields of gene and cellular therapy. Finally, in the future, it will be reasonable to think of individualized treatment depending on the genotype and other variables such as age or phenotype.

In conclusion, long-term outcome studies assessing efficacy and safety of GMP medical foods, BH4, LNAA supplementation and enzymatic therapy will be useful in providing the evidence allowing for standardization of management and will alternatively provide in a cost-effective way an individualized management plan for PKU patients.
